# Colonization screening positivity rates for novel multidrug-resistant organism healthcare containment responses during 2019–2022

**DOI:** 10.1017/ash.2023.373

**Published:** 2023-09-29

**Authors:** Danielle Rankin, Lucas Ochoa, Guillermo Sanchez, Kaitlin Forsberg, Meghan Lyman, Nijika Shrivastwa, Maroya Walters

## Abstract

**Background:** The CDC recommends a public health response when novel and targeted multidrug-resistant organisms (nMDROs), such as carbapenem-resistant organisms or *Candida auris*, are identified in healthcare settings in nonendemic areas. nMDRO responses are supported by healthcare-associated infection-antimicrobial resistance programs in 50 state and 6 local and territorial health departments. Annually, health departments report nMDRO responses to the CDC. We summarize nMDRO responses nationally and report our assessment of colonization screening positivity rates by healthcare setting and pathogen. **Methods:** We analyzed nMDRO response data reported by health departments for the period August 2019–July 2021; we excluded prevention efforts (ie, widespread screening based on facility-level risk factors). Among nMDRO responses in which colonization screening was performed, we calculated the proportion of responses in which screening detected additional cases of the index nMDRO and the colonization screening positivity, by healthcare setting and pathogen. **Results:** Among 2,051 nMDRO responses, 732 (36%) had ≥1 colonization screening (representing 44,845 colonization screenings), of which 24 (representing 17,467 colonization screenings) were prevention efforts and were excluded. Among the remaining 708 nMDRO responses, the healthcare setting most frequently included was acute-care hospitals (ACHs; 337 of 708, 48%); the least frequently included was long-term ACHs (LTACHs; 83 of 708, 12%). Carbapenem-resistant Enterobacterales were the most common index nMDRO prompting a response (408 of 708, 58%). Screening identified additional cases of the index nMDRO in 248 responses (35%) and 2,378 (9%) of 27,378 colonization screenings. Identification of the index nMDRO varied by pathogen and setting (Fig. 1). Overall, ventilator-capable skilled nursing facilities (vSNFs) were the facility type in which colonization screening most frequently identified additional cases of the index nMDRO (63 of 92 responses, 63%), and LTACHs had the highest colonization screening positivity (750 of 5,798, 13%). Similar colonization screening positivity was observed in ACHs (9%) and vSNFs (8%). On average, *Candida auris* and carbapenem-resistant *Acinetobacter baumannii* (CRAB) had the highest colonization screening positivity rates across all healthcare settings: CRAB, 493 (12.6%) of 3,907 screened; *Candida auris*, 1,344 (11.7%) of 11,466 screened (Fig. 1B). More than one-half of responses identified ≥1 case of the index nMDRO. **Conclusions:** During public health nMDRO responses, additional cases were regularly identified through colonization screening. Responses in vSNFs and LTACHs and to environmental pathogens like *Candida auris* and CRAB detected additional cases in more than one-half of responses, suggesting that spread commonly occurred prior to detection of the first clinical case. The use of colonization screening is an effective strategy to detect unidentified nMDRO colonization, especially in high-acuity postacute-care settings.

**Figure:**
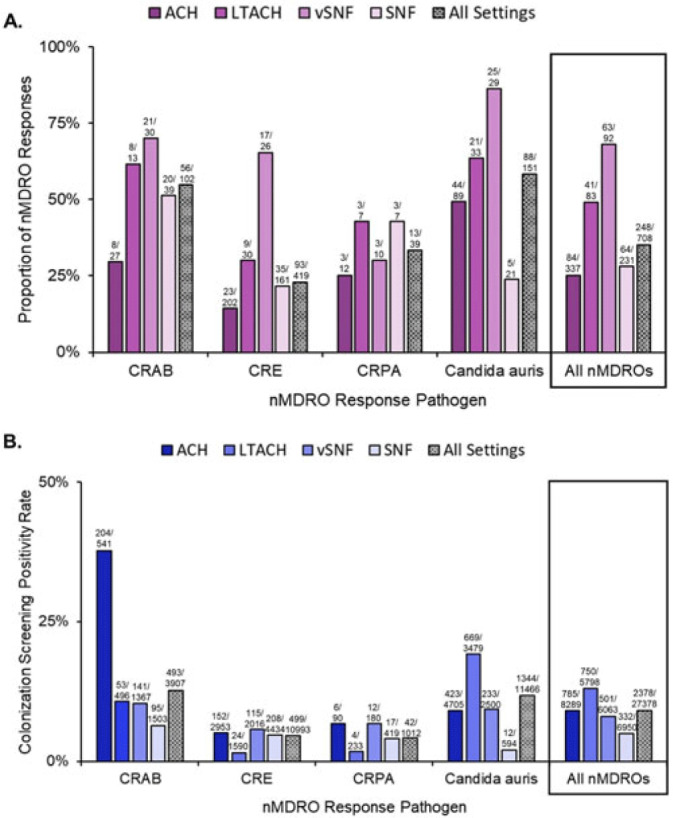

**Disclosures:** None

